# A Precise Segmentation Algorithm of Pumpkin Seedling Point Cloud Stem Based on CPHNet

**DOI:** 10.3390/plants13162300

**Published:** 2024-08-18

**Authors:** Qiaomei Deng, Junhong Zhao, Rui Li, Genhua Liu, Yaowen Hu, Ziqing Ye, Guoxiong Zhou

**Affiliations:** 1 College of Computer & Mathematics, Central South University of Forestry and Technology, Changsha 410004, China; 20210658@csuft.edu.cn (Q.D.); 20212864@csuft.edu.cn (R.L.); 2 Institute of Facility Agriculture, Guangdong Academy of Agricultural Sciences, Guangzhou 510640, China; zhaojunhong@gdaas.cn; 3 College of Electronic Information & Physics, Central South University of Forestry and Technology, Changsha 410073, China; genhualiu@csuft.edu.cn; 4 College of Computer, National University of Defense Technology, Changsha 410073, China; yaowenhu@nudt.edu.cn

**Keywords:** pumpkin seedling, point cloud, stem segmentation, CPHNet, CRA-MLP, PESA, HCE-dice loss

## Abstract

Accurate segmentation of the stem of pumpkin seedlings has a great influence on the modernization of pumpkin cultivation, and can provide detailed data support for the growth of pumpkin plants. We collected and constructed a pumpkin seedling point cloud dataset for the first time. Potting soil and wall background in point cloud data often interfere with the accuracy of partial cutting of pumpkin seedling stems. The stem shape of pumpkin seedlings varies due to other environmental factors during the growing stage. The stem of the pumpkin seedling is closely connected with the potting soil and leaves, and the boundary of the stem is easily blurred. These problems bring challenges to the accurate segmentation of pumpkin seedling point cloud stems. In this paper, an accurate segmentation algorithm for pumpkin seedling point cloud stems based on CPHNet is proposed. First, a channel residual attention multilayer perceptron (CRA-MLP) module is proposed, which suppresses background interference such as soil. Second, a position-enhanced self-attention (PESA) mechanism is proposed, enabling the model to adapt to diverse morphologies of pumpkin seedling point cloud data stems. Finally, a hybrid loss function of cross entropy loss and dice loss (HCE-Dice Loss) is proposed to address the issue of fuzzy stem boundaries. The experimental results show that CPHNet achieves a 90.4% average cross-to-merge ratio (mIoU), 93.1% average accuracy (mP), 95.6% average recall rate (mR), 94.4% F1 score (mF1) and 0.03 plants/second (speed) on the self-built dataset. Compared with other popular segmentation models, this model is more accurate and stable for cutting the stem part of the pumpkin seedling point cloud.

## 1. Introduction

As one of the important vegetable crops in China, pumpkin plays an important role in the agricultural field of our country. The phenotypic characteristics of pumpkin seedlings are crucial to the selection and production of pumpkin [1], including stem length, thickness and other parameters. By observing changes in the stem, the growth rate of pumpkin seedlings can be quickly assessed, the impact of current environmental conditions on pumpkin seedlings can be seen and the key opportunity for timely manual intervention and management can be provided. Continuous monitoring of crop stem information can help scientifically guide field management decisions. In practical application, the traditional measurement usually adopts manual measurement to obtain information on pumpkin seedling stems. Although manual measurement is the most direct and simple, the obtained trait data are relatively simple and the method consume a lot of time and human resources and prone to misdetection and leakage. In addition, manual measurement may cause a certain degree of damage to the pumpkin seedlings, which in turn affects the accuracy and consistency of the data. Especially in the case of large amounts of data, traditional manual measurement methods are obviously not efficient and applicable. Therefore, the development of efficient and accurate pumpkin stem partial cutting methods is crucial to improve the efficiency of pumpkin seedling phenotypic measurement and promote pumpkin breeding.

With the development of digital image [2,3] processing technology, visible light imaging has been widely used in the analysis of plant phenotypes [4]. The plant phenotype measurement method based on two-dimensional images [5,6,7] has been widely used in agricultural engineering [8,9,10] because of its advantages such as low equipment demand and easy operation. However, two-dimensional images are limited by imaging methods and cannot provide more spatial information; thus, they are powerless to deal with many parameters, such as stem thickness and root distribution [11] and other three-dimensional features. The three-dimensional point cloud is a type of data that can represent three-dimensional information, primarily containing depth information and compensating for the lack of depth in two-dimensional images. It largely avoids the problem of difficulty in accurately measuring phenotypes [12] due to single imaging angles. Xiao et al. [13] proposed a method of organ segmentation and phenotypic parameter measurement based on a three-dimensional point cloud of plants, which has a good segmentation effect on plants with mutually adhering leaves, and provides an effective solution for organ segmentation and phenotypic parameter measurement of multi-branch crops. Zhu et al. [14] proposed a maize point cloud stem and leaf segmentation method based on point cloud skeleton and optimal transmission distance. The average accuracy, average recall rate, average micro F1 score and average overall accuracy of stem and leaf segmentation were as high as 0.967, 0.961, 0.964 and 0.967, respectively. It provides an effective technique for high throughput phenotype detection of maize. Lin et al. [15] proposed an inter-field crop column spatial clustering segmentation method in order to achieve complete extraction and segmentation of individual plant objects in the field crop population point cloud data to complete automatic measurement of crop individual phenotypic parameters. Liu et al. [16] reconstructed three-dimensional point clouds of rapeseed plants based on multi-view stereo (MVS) methods, extending existing Euclidean distance and spectral clustering algorithms. They also utilized an iterative approach to achieve segmentation of rapeseed point cloud organs. Peng et al. [17] used the algorithm of Laplacian contraction to obtain the plant skeleton based on the single complete tomato [18] point cloud obtained by robots. After the skeleton was corrected, it was decomposed into sub-skeletons representing stems and leaflets, achieving segmentation between stems and leaf stalks. Then the leaves and petioles were separated by the MeanShift clustering method based on region growth. However, until now, there has been no research on the segmentation of stem and leaf by obtaining 3D point cloud data of pumpkin seedlings.

**Figure 1:**
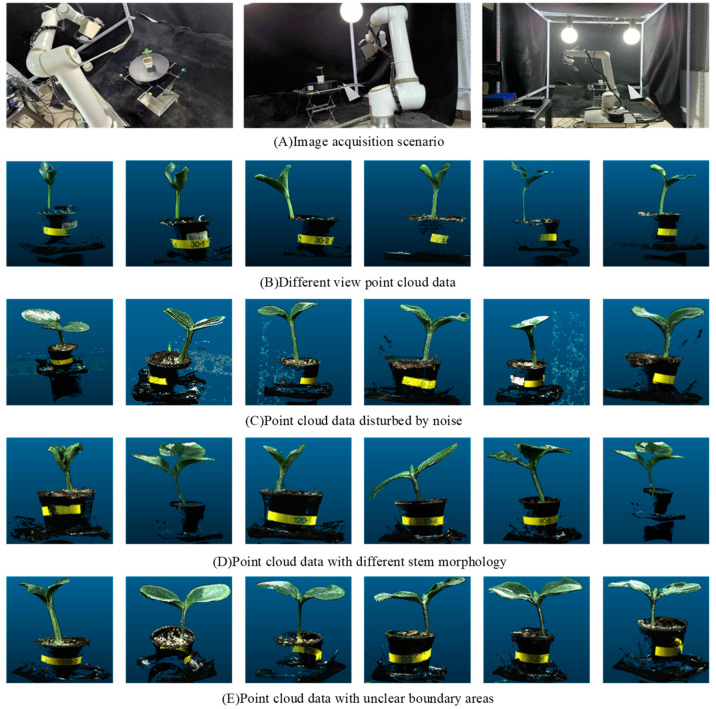
Point cloud data obtained from image acquisition scene and 3D reconstruction.

In recent years, with the development of deep learning technology, deep learning-based plant point cloud organ segmentation has become a feasible frontier research. Driven by large datasets, deep learning can extract a higher-dimensional basis without domain experts setting target data features, demonstrating powerful data processing advantages. In order to extract and analyze the phenotypic characteristic parameters of pumpkin seedlings more efficiently, accurately and objectively, this paper used deep learning technology to segment the pumpkin seedling stem according to the characteristics of the pumpkin seedling stem region, to measure and obtain the pumpkin seedling stem data more accurately. At present, there are the following problems in using deep learning technology to segment the stem of pumpkin seedlings: (1) Data annotation: There is a lack of an annotated 3D point cloud dataset of pumpkin seedlings. Currently, common 3D point cloud segmentation datasets include S3DIS, Stanford’s large-scale indoor scene RGB-D dataset [19], and KITTI, an outdoor large-scale point cloud dataset applied to automatic driving [20]. (2) Noise problem: Noise from the soil, leaf occlusion and collection point cloud data that is not related to pumpkin seedlings, such as wall and desktop, will interfere with the accuracy of segmentation, as shown in Figure 1C. (3) Multi-scale [21,22] problem: Pumpkin seedlings show rich morphological diversity, and their complexity is manifested in many differences in height, shape and other aspects. The stem morphology of pumpkin seedlings will be different due to other environmental factors such as sunlight during the growth stage [23], and multi-scale information needs to be processed, as shown in Figure 1D. (4) Unclear boundary area: The stem of pumpkin seedlings is closely connected with the potting soil and leaves, and the boundary is prone to blurring, as shown in Figure 1E. In view of this, we propose a new method: a model based on CPHNet for precise segmentation of pumpkin seedlings’ stems.

The contributions are as follows:

1. To enable the point cloud segmentation network to obtain abundant pumpkin seedling stem features, we constructed a pumpkin seedling point cloud dataset with accurate segmentation and labeling of pumpkin seedling stems for the first time, and applied classification labels to mark the pumpkin seedling stem and other areas.
2. To segment the stem of pumpkin seedlings more accurately, a partial cutting algorithm of pumpkin seedling stems based on point cloud data based on CPHNet was proposed. The design is as follows:
  (1) A Channel-Residual Attention Multi-Layer Perceptron (CRA-MLP) module is proposed to compress global spatial information and perform feature learning in the channel dimension, giving greater weight to the stem region of pumpkin seedlings, with residual connections enhancing the network’s ability to recognize and focus on the stem area while reducing the impact of background noise like soil and walls.
  (2) A Position-Enhanced Self-Attention (PESA) module is proposed to strengthen location information, enabling the model to understand the spatial relationships and geometric structure of pumpkin seedling point cloud data more accurately, while the self-attention mechanism helps the network dynamically adjust feature weights across different scales, improving adaptability and segmentation accuracy for the pumpkin seedling stem partial cutting task.
  (3) A Hybrid Cross Entropy and Dice Loss (HCE-Dice Loss) function is proposed, uniquely combining the classification accuracy of cross entropy, which requires high detail to help the model accurately classify the stem of the pumpkin seedling, with the boundary sensitivity of Dice loss, which aids the model in better identifying and segmenting stem details and boundary regions, thus allowing the training process to comprehensively consider pixel-level classification and target shape matching, ultimately improving the model’s segmentation accuracy and contrast for the pumpkin seedling stem boundary.
3. The CPHNet proposed in this paper achieves 90.4% mIoU, 93.1% mP, 95.6% mR and 94.4% mF1 on the self-built dataset. This method can effectively extract the stem features of pumpkin seedlings with different shapes, and realize the accurate segmentation of the stem region of pumpkin seedlings. By accurately segmenting the stem region of pumpkin seedlings, CPHNet can provide reliable data for further morphological analysis, growth trend prediction and other studies, and provide important support for healthy growth of pumpkin seedlings and disease prevention and control.

## 2. Materials and Methods

### 2.1. Data Acquisition

In this experiment, the “Zhuangshi” pumpkin (Cucurbita moschata D.) was used as the experimental material, which was purchased from Fujian Nongyou Seedling (China) Co., Ltd. (Fuzhou, China). The materials were cultivated in the artificial climate chamber of the Institute of Facility Agriculture, Guangdong Academy of Agricultural Sciences from July 2023 to August 2023. The pumpkin seeds are soaked in 55 °C hot water, followed by continued soaking after natural cooling for 4–6 h. The seeds are repeatedly washed upon completion and finally wrapped with disinfected wet gauze to keep them moist. They are placed in a constant temperature incubator at 28 °C to promote germination. When the seeds grow white radicle to 0.5 cm, they are neatly seeded in nutrient pots filled with substrate. The pumpkin material cultivation facility was in the artificial climate chamber (25 °C, the light time was 14 h during the day/10 h at night, the average relative humidity was 60–85% and the photosynthetically active radiation was 160 μmol m^−2^ s^−1^).

As shown in Figure 1A, the RVC-X-mini structural camera was installed on the end of the ELITE ROBOT six-axis robot arm, and the base of the robot arm and the position of the pumpkin seedling were fixed. Through the motion control of the robot arm, the camera was made to shoot around the pumpkin seedling from multiple angles to realize the multi-view point cloud data acquisition of the pumpkin seedling. The collected point cloud data were transformed and unified from different perspectives to the initial perspective through the transformation of the hand eye relationship and spatial coordinate matrix. Finally, through ICP iterative registration [24] and spatial filtering [25] processing, the three-dimensional reconstruction [26] of pumpkin seedlings was completed, and Figure 1B was obtained.

### 2.2. Data Processing

**Figure 2:**
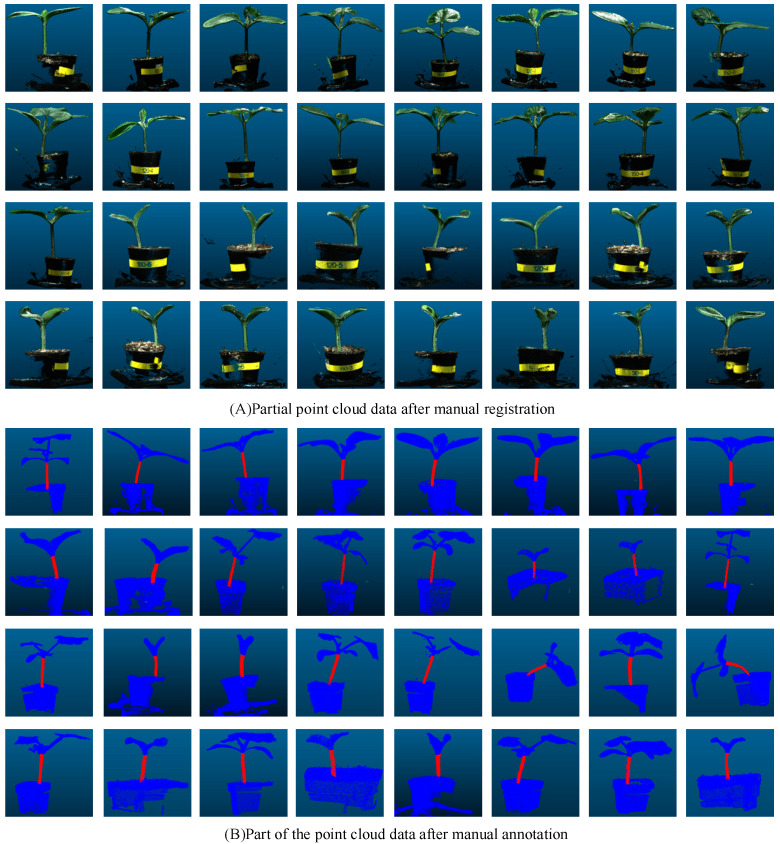
Point cloud data after registration and annotation.

In this study, 3D point cloud data of multiple pumpkin seedlings from different angles were obtained by laser scanning and input into the 3D point cloud processing software CloudCompare version 2.13.1 for manual accurate registration. The registered part is shown in Figure 2A. Subsequently, we used the method of artificial cropping to remove the points that had nothing to do with the pumpkin seedlings, such as the walls and tables. Given the limitations of the environment and acquisition equipment, point cloud data will inevitably be disturbed by noise. The causes of point cloud noise can be attributed to two types: outlier points and noise points [27]. The noise is mainly distributed around the stems and the potted plants, due to the reflection of the stems and leaves during the shooting process, and the occlusion of the connection between the soil and the stems. To remove these noise points and take into account the irregular density of point cloud data, statistical filtering method [28] was adopted to process the registered pumpkin seedling point cloud data to eliminate noise and outliers. The comparison of point cloud data before and after statistical filtering is shown in Table 1. Finally, we used the segmentation tool in the 3D point cloud processing software CloudCompare version 2.13.1 for manual annotation, separated the stem of the pumpkin seedling from other parts and labeled them separately, and the obtained point cloud data are shown in Figure 2B, thus obtaining a dataset containing 298 pumpkin seedling samples. Because the deep neural network model requires a large amount of data to extract effective features and avoid overfitting problems, the original dataset must be augmented by data enhancement [29,30]. The dataset in this paper is expanded by means of random rotation, random jitter and Gaussian noise [31]. The enhanced data are shown in Table 1. Finally, they are divided according to the ratio of 4:1, and the training set containing 952 pumpkin seedling point clouds and the test set containing 240 pumpkin seedling point clouds are obtained.

### 2.3. CPHNet

**Figure 3:**
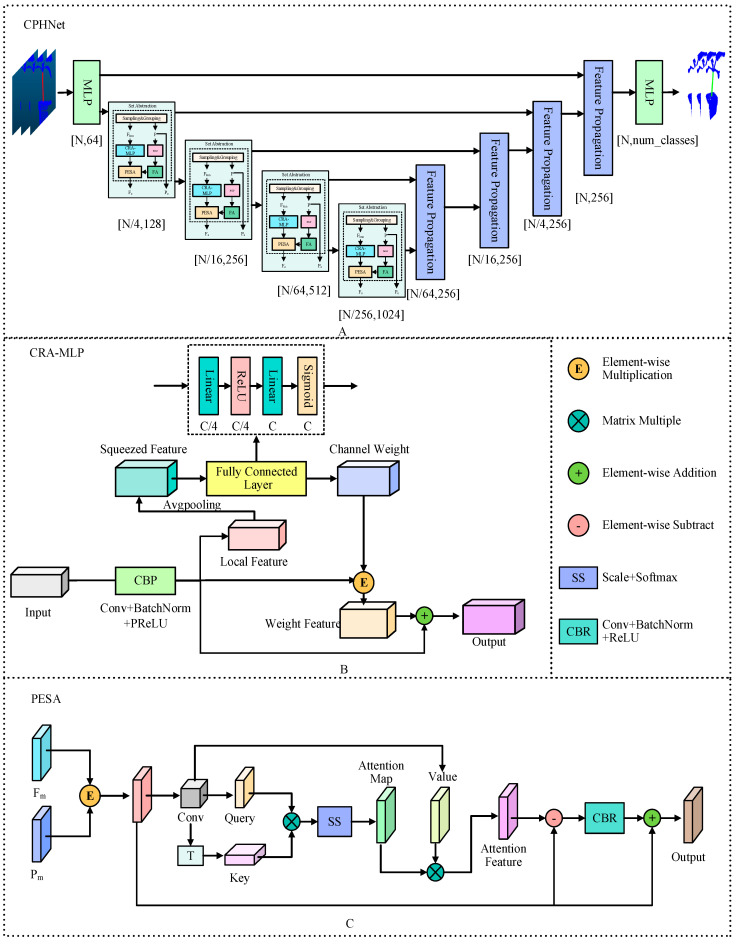
Structure of CPHNet.

According to the characteristics of the pumpkin seedling stem region, a point cloud data segmentation method of pumpkin seedlings based on PointNet++ [32], CPHNet, is proposed. The figure shows the overall network model structure. As shown in Figure 3A, it uses architecture similar to U-Net [33], with an encoder [34] and a decoder [35]. The input point cloud (N, D) first passes through a multilayer perceptron (*MLP*) layer to extract the features of each point. In this paper, the number of point clouds N is set to 4096, and the feature dimension D is set to 3. Four coding layers are then used to reduce the number of points while increasing the feature dimension of each point, the encoder part uses multiple set abstraction (SA) blocks for hierarchical sampling of the features of the point cloud, while the decoder part uses the same number of feature propagation blocks for step-by-step interpolation of the abstract features. The point cloud samples at a four-fold rate; that is, only 25% of the points are retained after each layer (N → N/4 → N/16 → N/64 → N/256), while increasing the feature dimension of each point (64 → 128 → 256 → 512 → 1024). After that, four decoding layers are used to restore the number of points in the point cloud to N. In the local feature-extraction stage, we proposed the CRA-MLP module as shown in Figure 3B, and replaced all the standard *MLPS* in the original backbone network. CRA-MLP is used to improve the network’s perception and differentiation of local features, so that it pays more attention to the features that are more representative of the current task, thereby reducing the chances of irrelevant features entering the feature fusion process and causing errors. In the global feature integration stage, we proposed PESA, as shown in Figure 3C. After adding it to local feature extraction, it dynamically adjusts the importance between features by learning the weight relationship between each feature and other features, to better capture the information and dependencies within. This allows the network to more accurately integrate global information based on the correlation between different features, thereby enhancing its ability to generalize. In the section on loss function, we proposed HCE-Dice Loss, which, compared with the original standard cross-entropy loss function, can focus on both pixel-level classification and target shape matching, which means that the model can better learn the accurate segmentation of different parts, and can provide more comprehensive feedback during training. Algorithm 1 lists the steps of the CPHNet point cloud segmentation algorithm, with undefined variables described in detail in the following sections.

**Algorithm** **1:** All experiments in this paper used the default values.

∂∈(1e−3,1e−5),m=0.9,n=150,batch size=8

**Require**: ∂, the learning rate. m, the momentum. n, the number of epochs. θ, CPHNet parameter.1: **while** θ has not converged:2: **for** epoch = 0, …, n **do:**3: Randomly select a batch of data from the training set ${\left\{x^{\left(i\right)}\right\}}_{i=1}^{batch\;size}$4: $\phi =SG(knn(x^{\left(i\right)}))$ ϕ is local features.5: **CRA-MLP**6: $F_{l}=MLP(\phi )$7: $F_{CRA-MLP}=CPR(F_{l})$8: **PESA**9: $P_{o}=FA(MLP(P_{i}))$ $P_{i}$ is position information.10: $F_{o}=PESA(F_{CRA-MLP},P_{o})$11: $g_{w}\leftarrow \nabla_{\theta }(w_{c}\times L_{ij}^{Cross\;Entropy}+w_{d}\times L_{ij}^{Dice})$ $w_{c}$, the weights of Cross Entropy Loss. $w_{d}$, the weights of Dice Loss. $g_{w}$ is the grad during the training process.12: $\theta \leftarrow \theta +\partial \cdot Adam(g_{w},m)$13: **end for**14: **end while**

#### 2.3.1. Channel Residual Attention Multilayer Perceptron

In the pumpkin seedling stem region segmentation task, some noise generated by potting soil and photographing point cloud data often has a great impact on the point cloud segmentation network. In the PointNet++ model, *MLP* [36] is a multi-layer neural network, whose function is to carry out nonlinear transformation and a combination of the features of each point, so as to extract richer and more representative features. The original feature of each point is mapped by multi-layer nonlinear mapping of *MLP*, and a new high-dimensional feature representation is obtained. This process enables the model to better understand the local features of each point and improve the recognition ability of complex objects. However, the effect of reducing noise is still small. In addition, when the stem area of pumpkin seedlings is separated, because the stem area is closely connected to the leaves of pumpkin seedlings and the potting soil, the segmentation network model usually mistakes some soil and leaves for the stem part of pumpkin seedlings. We then came up with the idea of adding an attention mechanism [37,38] to the *MLP*, which in deep learning methods can be used to suppress this background noise and direct the network’s emphasis on key target areas. Attention mechanisms have been extensively studied and have been shown to significantly enhance the performance of visual neural networks. SE [39] enhanced the focus on channel dimensions and modeled the importance of each feature channel. Then, according to the usefulness of each channel, different weights are assigned to each feature to suppress or emphasize the expression of the feature channel. This not only enhances the feature representation capability of the network, making the network more focused on extracting and utilizing the feature information useful for the current task but also has the robustness to the noise input. It is of practical significance to reduce the effect of noise on the segmentation of pumpkin seedling stems.

Considering the complexity and the importance of feature information to the point cloud segmentation model, we added residual connection [40] to SE, which can help information transfer in the network, especially after the channel attention mechanism. Even if the channel attention mechanism reduces the weight of some channels, the information on the original features can still be retained through the residual connection to prevent the loss of information. Residual connections also help mitigate the problem of disappearing gradients, especially in deep networks such as point cloud segmentation. It makes it easier for gradients to propagate back to earlier layers of the network, thus ensuring the stability and efficiency of training. It can make the network deeper and improve the expressiveness of the model. This allows the network to better adapt to complex tasks and data distribution. In addition, we also optimize the nonlinear elements in *MLP* to improve the performance and robustness of the model. The modified *MLP* is named CRA-MLP in Figure 3B.

The improvement of CRA-MLP consists of two parts:

1. For the nonlinear mapping layer in the *MLP* of the original model, we used the PReLU [41] activation function instead of the standard ReLU [42] activation function to improve the performance and training stability of the network. Unlike ReLU, PReLU allows some neurons to have negative activation values, thus providing a degree of linear responsiveness. This allows PReLU to learn more complex features. ReLU has a “dead neuron” problem [43]; that is, some neurons may never be activated during training, resulting in weights that are not updated. PReLU can mitigate this problem to some extent because it allows a subset of neurons to remain non-zero activated. And the parameters of PReLU can be trained, which means that the network can adaptively learn the activation function of each neuron, resulting in a stronger fitting ability.

PReLU’s calculation formula is as follows:

(1) $$f(x)=\left\{\begin{array}{lll} x, & & x\geq 0\\ \alpha x, & & x<0 \end{array}\right.$$

2. Add channel residual attention mechanism at the end of *MLP*.

The CRA-MLP consists of the following:

Firstly, the locally sampled point cloud is processed through convolution, normalization and PReLU nonlinear activation to obtain feature map $F_{D}$. We performed global average pooling along the height and width dimensions of the feature map in the channel direction to obtain the tensor along that channel. The tensor learns the weight information between channels through the FC fully connected layer to improve the feature information quality of the feature graph. Then, the Sigmoid activation function [44] is used to map the output of the fully connected layer as the weight coefficient ω. These weight coefficients are then applied to the input feature map. This step multiplies the feature value of each channel by its corresponding weight coefficient, thus making the network pay more attention to the important channel features during the learning process. Finally, the weighted input feature map is added to the original input feature map, and the output after the channel attention mechanism is obtained by residual connection.

The above process is represented by the following formula:

(2) ω=σW2⊗ReLUW1⊗AvgPoolFD

(3) $$output=F_{D}⊙\omega .\exp end\_as\left(F_{D}\right)+F_{D}$$

In the above formula, $W_{1}\in R^{\left(C\times \frac{C}{4}\right)}$ and $W_{2}\in R^{\left(\frac{C}{4}\times C\right)}$ are two weight matrices, respectively, representing linear transformations in full connection, σ represents Sigmoid activation function, AvgPool represents global average pooling, ⊗ represents matrix multiplication, ⊙ represents element-wise multiplication operation.

The section on the effectiveness of CRA-MLP discusses its experimental study.

CRA-MLP effectively reduces the sensitivity of the model to noise by adding the channel residual attention mechanism within the multi-layer perceptron.

#### 2.3.2. Self-Attention Mechanism of Position Enhancement

Pumpkin seedlings show rich morphological diversity, and their complexity shows many differences in height, shape and so on. The stem shape of pumpkin seedlings will be different due to other environmental factors such as sunlight during the growth stage, which also leads to the phenomenon of missing or increasing the number of separated stems during segmentation. The self-attention mechanism [45,46,47] can adapt to features at different scales, and can help models model features at different levels, so as to improve the model’s ability to understand local and global information, so as to effectively reduce the occurrence of this situation.

Self-attention was initially predominantly applied in the natural language domain. Vaswani et al. [48] employed self-attention mechanisms to construct the earliest transformer, primarily for machine translation purposes. With the deepening of research, the self-attention mechanism has been applied to the field of computer vision. Dosovitskiy et al. [49] prove that transformer can be applied to 2D images. Then Engel et al. [50] designed point transformer and achieved great success in the 3D field. In the Self-Attention Layer (SA) of PCT [51] proposed by Tsinghua University, the input features and location information are fully utilized. Through query, key and value transformation, the model can dynamically calculate the attention weight between each pair of points. This implies that the significance of each point is dynamically determined based on the actual input data and positional information, rather than being predefined or reliant on specific patterns. This enables the model to flexibly comprehend global relationships and local structures in point cloud processing, thereby enhancing the model’s performance and robustness. However, in this self-attention layer, only the incoming feature information is emphasized, while the positional information is simply passed through a convolution layer before being added to the feature information. This approach does not fully utilize the positional information.

Considering the importance of location information in point cloud segmentation [52], we optimized the simple convolution operation of location information in SA to make it pass through a multi-layer perceptron, and the location information can pass through multiple convolution layers, batch normalization layers and activation functions, thus improving the processing ability of the model for location information. Then it passes through an FA feature attention layer to help the network better select the features of the location information. Finally, the addition operation of the feature information and the location information is changed to the multiplication operation, so that the location information has an impact on each feature dimension, to integrate the location information and the feature information more effectively. The modified SA is named PESA in Figure 3C.

PESA consists of two parts:

1. Enhancement of coordinate position information. First, the 3D original coordinate information P is passed through the *MLP*, and the convolution, normalization and nonlinear activation are carried out in the *MLP*. Through convolution, the channel number of the coordinate information matches the number of the incoming characteristic channel, and the position coordinate information is enriched to obtain $P_{i}$. Then $P_{i}$ is weighted by the FA feature concern layer to get $P_{o}$, which helps the network to better select the features of the location information. FA is a simple feature of concern.

The above process can be expressed by the following formula:

(4) $$P_{0}=P_{i}⊙\sigma \left(MLP\left(Sum\left(P_{i}⊗P^{T}{\;}_{i}\right)\right)\right)$$

In Formula (4), σ stands for Sigmoid activation function, T stands for transpose, Sum stands for the sum of all rows to the first row, *MLP* is a multi-layer perceptron, which includes convolution, BN normalization and Relu activation function.

2. Self-attention weighting. Firstly, the incoming enhanced position information $P_{o}$ and feature information $F^{i}$ are multiplied element by element to obtain the fused feature $F^{\prime }$, where Q, K and V are the matrix generated by the linear transformation of the fused feature $F^{\prime }$, and the process is defined as follows:
  (5) $$F^{\prime }=P_{o}⊙F_{i}$$
  (6) $$\left(Q,K,V\right)=F^{\prime }⊗\left(W_{q},W_{k},W_{v}\right)$$

In Formula (6), where $W_{q}\in R^{\left(C\times \frac{C}{4}\right)},W_{k}\in R^{\left(C\times \frac{C}{4}\right)},W_{v}\in R^{\left(C\times C\right)}$ are three weight matrices, respectively.

Then we used Q matrix and K matrix to get attention weight E through matrix dot product, and then normalized attention weight to get attention weight A. The process is defined as follows:

(7) $$E=Q⊗K^{T}$$

(8) $$a_{i,j}=soft\max(E)$$

(9) $$A=\frac{a_{i,j}}{1e-9+\sum_{k}a_{i,k}}$$

Next, the attention weight A is weighted with the value matrix V to obtain the output $F_{sa}$ of the self-attention mechanism. Then, output $F_{sa}$ is used to calculate the offset from the input feature $F^{\prime }$ through element-wise subtraction. The resulting outcome is passed into the CBR network. Finally, a residual connection is established between this output and the input features $F^{\prime }$ to preserve the original information and introduce the adjusted details, producing the ultimate output features $F_{o}$. The process is defined as follows:

(10) $$F_{sa}=A⊗V$$

(11) $$F_{o}=CBR(F_{sa}-F^{\prime })+F^{\prime }$$

In Formula (11), CBR indicates that it is processed by convolutional layers, batch normalization and ReLU activation function operations.

By using the location-enhanced attention mechanism, PESA enables the model to adapt to different degrees of feature information, and enhances the applicability of the model to different forms of pumpkin seedling point cloud data.

#### 2.3.3. Hybrid Loss Function of Cross Entropy Loss and Dice Loss

The stem of the pumpkin seedling is closely connected with the potting soil and leaves, and the boundary of the stem is easy to be blurred. A single cross-entropy loss function tends to pay more attention to class accuracy and tends to favor most classes. The Dice loss function, on the other hand, focuses on the geometric matching of the target, which helps the model to capture the contour and boundary information of the target more accurately, but it may ignore the classification accuracy at the point level. Therefore, it is necessary to combine cross entropy with Dice loss function. In this way, the model can consider the accuracy of the class and the matching of the target shape, so as to solve the problem of fuzzy stem boundary.

Cross entropy loss [53,54] is a loss function widely used in classification tasks. For the point cloud semantic segmentation task, its mathematical definition is as follows:

(12) $$ce\_loss=-\frac{1}{N}\sum_{i=1}^{N}\sum_{C=1}^{C}y_{i,c}\log\left(p_{i,c}\right)$$

In Formula (12), N is the total number of points, C is the number of classes, $y_{i,c}$ is the true label that the i th point belongs to class c, $P_{i,c}$ is the probability that the point predicted by the model belongs to class c.

Cross entropy loss has a strong optimization performance in point cloud semantic segmentation task, which can effectively punish classification errors and promote rapid convergence of the model. However, when faced with a situation of class imbalance, cross entropy loss may lead to performance degradation on a few classes.

Dice loss [55,56] is a loss function designed for segmentation tasks, measuring similarity by computing the overlap between predictions and targets. Its mathematical definition is as follows:

(13) $$dice\_loss=1-\frac{2\times \mathrm{intersection}+\varepsilon }{\mathrm{pred}\_\mathrm{sum}+\mathrm{target}\_\mathrm{sum}+\varepsilon }$$

In Formula (13), intersection is the intersection of the predicted and target values, pred_sum and target_sum are the sum of the predicted and target values, respectively, and ε is a smooth term to avoid dividing by zero.

Dice loss is a loss function for splitting tasks that measures similarity by calculating how much overlap there is between the prediction and the target. Dice Loss is more tolerant of small errors than cross entropy loss, and thus may be more robust in the face of noise or incomplete data. However, Dice loss may also encounter numerical instability issues, necessitating appropriate parameter tuning to ensure training stability.

Considering that in the task of pumpkin seedling stem region segmentation, accurate classification and precise segmentation of the stem area are both crucial. In order to make full use of the advantages of cross entropy loss and Dice loss, we proposed a hybrid loss function HCE-Dice Loss, which combines cross entropy loss and Dice loss. The formula is defined as follows:

(14) HCE - Dice Loss=weight_ce×ce_loss+weight_dice×dice_loss

In Formula (14), weight_ce and weight_dice are the weight parameters used to balance the cross entropy loss and Dice loss.

Hybrid cross entropy and Dice loss make full use of the advantages of both, balance the accuracy of classification and segmentation, and improve the comprehensive performance of the model. Cross entropy loss pays attention to classification accuracy, while Dice loss pays attention to segmentation accuracy. Mixing the two can ensure classification accuracy while improving segmentation accuracy. By adjusting the weight parameters of cross entropy loss and Dice loss in mixed loss, the relative importance of the loss function can be flexibly controlled to adapt to different task requirements.

## 3. Experimental Results and Analysis

### 3.1. Environment and Settings

To avoid the influence of different experimental conditions on the results of CPHNet, all experiments in this study are conducted in the same hardware and software environment. The NVIDIA GeForce RTX 3060 GPU and AMD R7 5800H CPU are the primary hardware used in this experiment. Although the Python and CUDA versions do not affect the experimental results, they have to be compatible with specific software and hardware. CPHNet is built using PyTorch 2.0.0 and Python 3.9.13. Table 2 provides specific hardware parameters.

**Figure 4:**
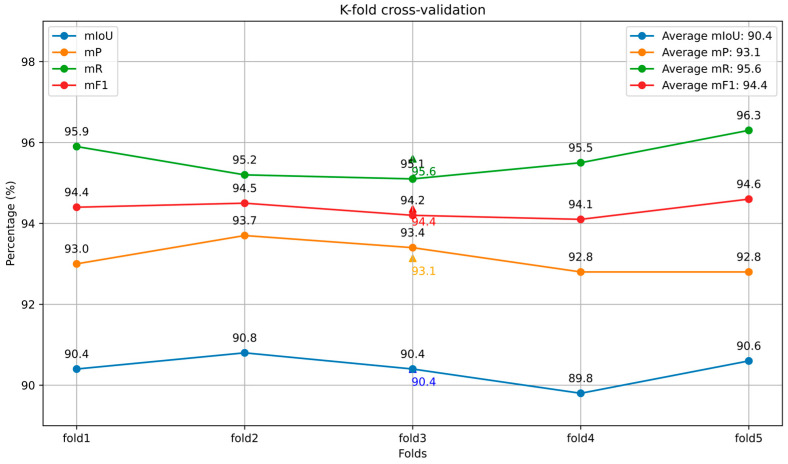
The results of k-fold cross-validation.

Before inputting the data, the dataset is augmented by random rotation, adding random jitter, and introducing Gaussian noise, resulting in a total of 1192 pumpkin seedling point cloud datasets. In this study, we conducted 5-fold cross-validation for training to properly assess the model’s performance while avoiding overfitting. The results are shown in Figure 4. The dataset is divided into a 4:1 ratio, yielding a training set containing 952 pumpkin seedling point clouds and a test set containing 240 pumpkin seedling point clouds. The training set is used to train the model’s parameters and weights, while the test set is used to evaluate the model’s performance and generalization ability.

During the training phase, the batch size for network training is set to 6, with an initial learning rate of 0.001. The training iteration number, or epochs, is set to 150, with the maximum and minimum learning rates set at $1\times 10^{-3}$ and $1\times 10^{-5}$, respectively. The optimizer used is Adam with a momentum of 0.9.

### 3.2. Evaluating Indicator

In this paper, we employed the mIoU metric, mP (mean precision), mR (mean recall), mF1 (mean F1 score), parameter size Params and speed as evaluation metrics for assessing the model’s performance.

mIoU is a commonly used evaluation metric for semantic segmentation tasks, which comprehensively considers the Intersection over Union (IoU) for all categories and provides an overall assessment of the model’s performance in multi-class segmentation tasks. The relevant formula is as follows:

(15) $$mIoU=\frac{1}{N}\sum_{i=1}^{N}\frac{TP_{i}}{TP_{i}+FP_{i}+FN_{i}}$$

where N represents the total number of categories, $TP_{i}$, $FP_{i}$ and $FN_{i}$, respectively, denote the true positives, false positives and false negatives for the i-th class.

The mIoU computes the average IoU for each category, providing a comprehensive global performance measure that accurately reflects the model’s segmentation effectiveness across various categories. Utilizing mIoU allows us to holistically assess the model’s performance in each category rather than merely focusing on individual category accuracy. In our experiments, we compared the model’s predictions on the test set against the ground truth labels to calculate the IoU for each category, eventually obtaining the numerical value of mIoU.

The mP (mean precision) is utilized to gauge the accuracy of the model in the pumpkin seedling stem segmentation task. It is defined as the average precision across all categories, with the relevant formula as follows:

(16) $$mP=\frac{1}{N}\sum_{i=1}^{N}\frac{TP_{i}}{TP_{i}+FP_{i}}$$

where N represents the total number of categories,$TP_{i}$ is the true positives for the i-th class and $FP_{i}$ is the false positives for the i-th class.

The mR (mean recall) is employed to measure the comprehensiveness of the model in the pumpkin seedling stem segmentation task. It is defined as the average recall across all categories, with the relevant formula as follows:

(17) $$mR=\frac{1}{N}\sum_{i=1}^{N}\frac{TP_{i}}{TP_{i}+FN_{i}}$$

where N represents the total number of categories, $TP_{i}$ is the true positives for the i-th class and $FN_{i}$ is the false negatives for the i-th class.

The mF1 (mean F1 score) comprehensively combines mean precision and mean recall, serving as a holistic evaluation metric. It balances accuracy and comprehensiveness by utilizing the harmonic mean, with the relevant formula as follows:

(18) mF1=1N∑i=1N2×Precisioni×RecalliPrecisioni+Recalli

To evaluate the real-time performance of the model, we employed the speed metric (Speed). In this paper, it represents the average number of point clouds segmented per second. The formula for calculating Speed is as follows:

(19) $$Speed=\frac{1}{t}$$

where t represents the time required to segment each point cloud data.

To measure the model’s complexity, we introduced the number of parameters (Params) as an evaluation metric. The number of parameters refers to the total count of weights and biases that the model needs to learn during the training process.

### 3.3. CPHNet Performance Analysis

**Figure 5:**
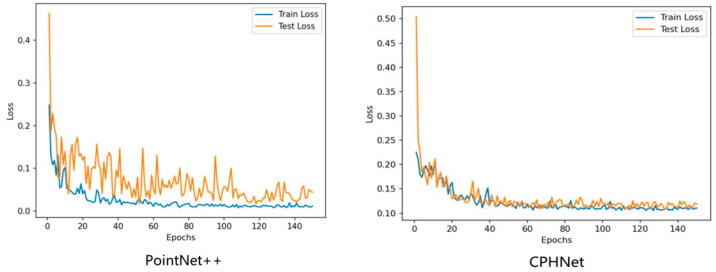
Change curve of the loss rate.

To validate the effectiveness of CPHNet on PointNet++, we conducted a series of performance evaluations on the test set. To comprehensively analyze our network model, we conducted experimental assessments using various metrics. The results demonstrate a significant improvement of CPHNet over PointNet++ in metrics such as mIoU, mP, mR and mF1. This superiority can be attributed to the outstanding performance of CRA-MLP in extracting local features, accurately capturing micro-details in the data and effectively reducing noise interference. Additionally, the self-attention mechanism introduced in the PESA module enhances the integration capability of global features, further improving the segmentation accuracy of pumpkin seedling stems. Furthermore, we compared the differences between the two models during the training process, and the change curve of their loss rates is shown in Figure 5. It was observed that the fluctuation in training and testing loss rates of CPHNet is smaller, with a smaller difference between training and testing losses, and a more stable final convergence point, further confirming its better alignment with the characteristics of pumpkin seedling stems. In contrast, despite the lower loss function value at the convergence point for PointNet++, its testing loss demonstrates significant instability, indicating a certain bias in its adaptability to the test and training sets. These comprehensive results highlight the superiority of the CPHNet model in the segmentation task of pumpkin seedling point cloud stems.

### 3.4. Module Effectiveness Experiments

To evaluate the effectiveness of data preprocessing, the CRA-MLP module, the PESA module and the HCE-Dice Loss within our model, we conducted a series of experiments for illustration. This section aims to explore the impact of each module on the model’s performance and present the experimental outcomes under various settings.

#### 3.4.1. Effectiveness of the Data Processing

In this paper, we performed statistical filtering and data augmentation on the original pumpkin seedling point cloud data. To validate their effectiveness, we conducted comparative experiments on these three data aspects, the results are detailed in Table 3.

The experimental results indicate that the statistical filtering effectively reduced outliers in the data, thereby enhancing various metrics: mIoU increased by 2.2%, mP by 3.1%, mR by 0.9% and mF1 by 1.2%. Through data augmentation, the model encountered a wider range of sample variations, leading to a better understanding of features and changes under diverse circumstances. This improved the model’s ability to generalize to the test dataset, enhancing its overall generalization capacity, along with improvements in all metrics. Subsequent experiments were conducted based on the data preprocessing.

#### 3.4.2. Effectiveness of CRA-MLP

In this study, we opted to incorporate the CRA-MLP module instead of the conventional *MLP* in PointNet++. As depicted in Table 3, CRA-MLP introduces additional parameters, which to some extent, increase the model’s complexity. However, this enhancement notably improves the accuracy of segmenting the pumpkin seedling stem region, elevating mIoU by 4.5%, alongside improvements in other metrics. It is noteworthy that with the introduction of more parameters, there is a slight decline in the model’s inference speed. Nevertheless, considering the enhancements in mIoU, mP, mR and mF1, this speed reduction can be deemed acceptable.

Furthermore, we replaced the traditional ReLU activation function in *MLP* with the PReLU activation function. To evaluate the superiority of PReLU over ReLU, a series of experiments were conducted, and the results are presented in Table 3. The experimental outcomes demonstrate that the PReLU activation function improves the model’s performance to some extent without introducing additional parameters, while the inference speed remains relatively unchanged. Compared to the traditional ReLU activation function, adopting the PReLU activation function led to an increase in mIoU by 0.6%, mP by 0.7%, mR by 3.1% and mF1 by 1.8%.

In summary, the addition of the CRA-MLP module effectively enhanced the model’s performance.

#### 3.4.3. Effectiveness of PESA

In this experiment, we introduced the PESA module, an enhancement built upon SA (Self-Attention) by emphasizing the significance of positional information and integrating it into the model’s global feature integration phase. This enhancement of positional information involves *MLP* processing of incoming positional data and the application of the FA (Feature Aggregation) feature enhancement layer. We validated the superiority of the PESA module through experimental comparisons, as shown in Table 3.

Two significant findings emerged from the experimental results: Firstly, the introduction of self-attention mechanisms in the global feature integration stage proved effective. Despite the introduction of additional parameters and a slight sacrifice in inference speed, the model demonstrated a 1.8% increase in mIoU, a 0.1% increase in mP, a 3.1% increase in mR and a 1.5% increase in mF1, thereby enhancing segmentation accuracy. Secondly, the strategy for enhancing positional information was effective. Compared to the standard SA mechanism, PASA processed positional information through *MLP* and then underwent FA feature enhancement, strengthening the representation of positional details. This approach effectively utilized critical positional information within the pumpkin seedling point cloud data, enhancing the model’s generalization ability and further improving the indicators. It is noteworthy that this process did not introduce additional parameters, and the inference speed remained at a comparable level.

#### 3.4.4. Effectiveness of HCE-Dice Loss

In the loss function aspect, we replaced the single cross-entropy loss function in PointNet++ with HCE-Dice Loss. To validate the superiority of HCE-Dice Loss, we conducted comparative experiments, and the results are presented in Table 3.

The experimental results indicate that compared to a single cross-entropy loss function, HCE-Dice Loss demonstrates improvements in mIoU, mP, mR and mF1, especially with the largest increase observed in mR by 7.8%. This enhancement signifies that HCE-Dice Loss effectively improves the matching of pumpkin seedling stems, enabling the model to more accurately identify more target objects.

#### 3.4.5. Ablation Experiment

In order to verify the validity of the PointNet++ based method proposed in this paper, we conducted a series of ablation experiments on the CPHNet architecture, and the detailed results are shown in Table 4.

During the experimental process, we utilized the control variable method to sequentially add CRA-MLP, PESA and HCE-Dice Loss and then conducted 8 groups of ablation experiments on these 3 modules in combination with CPHNet. By comparing the experimental results, we observed that CRA-MLP exhibited the best overall performance, displaying significant improvements across all metrics without adding an excessive number of parameters. Contrasting the results between groups F and H, we noticed a 3.4% improvement in mIoU and the largest enhancement in mP. This outcome effectively demonstrates that the PESA module can efficiently integrate global features, thereby significantly improving the model’s segmentation accuracy. Furthermore, comparing the results between groups E and H confirmed that HCE-Dice Loss effectively enhances the model’s segmentation accuracy without introducing additional parameters. In conclusion, CPHNet achieved a 10.5% improvement in mIoU, 8.6% in mP, 5.8% in mR and 7.4% in mF1 compared to the traditional PointNet++ method. However, the addition of modules introduced more parameters, leading to a slight decrease in the model’s inference speed. Meanwhile, the results of eight sets of experiments show that CRA-MLP, PESA and HCE-Dice Loss are all effective at enhancing the model’s segmentation accuracy. Thus, we can conclude that CPHNet outperforms the PointNet++ model in the task of pumpkin seedling point cloud stem segmentation.

To visually assess the effectiveness of each module in CPHNet for segmenting pumpkin seedling point cloud data, we compared the PointNet++ model and successively added the CRA-MLP, PESA and HCE-Dice Loss modules. We compared the segmentation visualization results for three representative pumpkin seedling point cloud datasets. The visualization results are shown in Table 5. In the visualization images, the pumpkin seedlings are classified into two categories: the green area represents the stem, while the blue area represents other parts.

In Group A, we selected the segmentation results of pumpkin seedlings with multiple branches to evaluate the performance of the network on different morphologies of pumpkin seedlings. The results show that PointNet++ incorrectly labeled some branches as stems, and the segmentation of stems is incomplete. After the addition of CRA-MLP and PESA modules, the false detection rate is reduced and the integrity rate is significantly improved. This improvement can be attributed to the fact that the self-attention mechanism in PESA helps model feature modeling at different levels, enhances the understanding of local and global information, makes the model better adapt to different shapes of pumpkin seedlings and effectively reduces misjudgments. In addition, the addition of CRA-MLP enhances local feature extraction and provides a more flexible acceptance domain, thus improving the perception and segmentation integrity of stem features. The subsequent use of HCE-DiceLoss further improved the segmentation accuracy, resulting in smoother edges of the segmentation output.

In Group B, we tested segmentation performance using pumpkin seedlings with multiple potting containers, aiming to simulate the presence of disturbing objects. The results show that both PointNet++ and CPHNet can maintain good stem integrity, but PointNet++ mislabels most of the soil as stems, indicating that it has poor treatment of soil noise, while CPHNet can accurately segment stems. This difference is attributed to the CRA-MLP module in the CPHNet, whose internal residual connections effectively reduce the loss of feature information, allowing the model to more accurately distinguish the fine boundaries of plant stems and soil. In addition, CRA-MLP learns complex features through a multi-layer perceptron network, significantly reducing the interference of soil noise on stem segmentation. The comprehensive properties of the CRA-MLP allow CPHNet to more precisely separate plant stems from the soil, enhancing the segmentation accuracy and robustness of pumpkin seedling point cloud data for stem identification.

In Group C, we tested pumpkin seedling point cloud data containing various noises, such as walls and outliers, to evaluate the model’s segmentation performance in complex scenarios. The results show that PointNet++ is susceptible to noise and mismarks outliers and container parts as stems. After the integration of CRA-MLP, its attention mechanism and multi-layer sensor effectively filtered out the environmental noise, but due to the short stem length in this pumpkin seedling point cloud data, some leaves in the stem and leaf connection area were still misjudged as stems. With the integration of PESA and HCE-DiceLoss modules, the situation has improved significantly. PESA enhances the ability of the model to process global and local features, and improves the robustness of stem segmentation. The HCE-Dice Loss module, by combining Dice loss and cross entropy loss functions, further strengthened the network’s ability to accurately differentiate stems, resulting in smoother segmentation edges.

In Group D, we tested point cloud data containing two pumpkin seedlings at the same time to evaluate the model’s segmentation performance in a multi-plant scenario. The results showed that PointNet++ could only separate a small part of the stem of two pumpkin seedlings, while CPHNet could completely separate the stem of two pumpkin seedlings. This is because the channel attention mechanism in CRA-MLP adaptively adjusts the weight of each channel according to the different characteristics of different pumpkin seedlings, effectively distinguishing and dividing the stems of each pumpkin seedling. In addition, PESA’s self-attention mechanism helps the model better understand the relationships between points in the point cloud data, enhancing the model’s ability to segment multiple pumpkin seedling stems simultaneously.

Based on the comparative experimental results, it is evident that CPHNet outperforms PointNet++ in terms of performance. CPHNet not only enhances the accuracy of segmenting pumpkin seedling point cloud data stems but also effectively mitigates issues related to false detections and stem discontinuity caused by environmental noise and the diverse morphology of pumpkin seedling stems. This further improves the segmentation model’s robustness and applicability. Additionally, it effectively addresses situations where the stem edges appear blurry, resulting in smoother segmentation edges.

### 3.5. Comparison of State-of-the-Art (SOTA) Models

In the same testing environment and dataset, we conducted a comparative analysis of CPHNet against six point cloud segmentation networks: PointNet [57], PointNet++, PointNet++(MSG) [32], GACNet [58], PointNeXt [59] and CSANet [60]. Table 6 displays the test results.

In the point cloud segmentation models, PointNet stands as the initial neural network architecture capable of directly processing unordered point clouds, endowed with end-to-end processing capabilities, thus circumventing the intricate preprocessing steps in traditional methods. Nonetheless, PointNet lacks local feature fusion, resulting in relatively weaker feature-extraction capabilities. Building upon this foundation, PointNet++ introduces further enhancements by incorporating a ‘Set Abstraction’ module, enabling a more precise capture of both local structures and global information within point cloud data. Consequently, it demonstrates heightened classification and segmentation performance, particularly in complex scenarios. This elucidates the significant reason for selecting PointNet++ as the foundational network. PointNet++(MSG) increases the number of feature points by employing multi-scale grouping to concatenate features, thereby mitigating the influence of sample point quantity and further enhancing segmentation performance. In employing attention mechanisms, GACNet utilizes the graph attentional convolutional algorithm, capable of computing edge weights between the central point and each neighboring point, thereby improving the network’s performance at the segmented edges. PointNeXt excels in segmentation by hierarchically learning features and integrating a Point Attention Transformer (PAT) for dynamic feature adjustment, enhancing accuracy and efficiency in processing complex point cloud data. CSANet adopts a cross self-attention mechanism, interactively extracting coordinate and feature information to enhance the model’s feature-extraction capabilities, subsequently improving segmentation performance. Based on the experimental results, our proposed model, CPHNet, outperforms the other six models across various metrics such as mIoU, mP, mR and mF1. Compared to the base network, PointNet++, our model shows improvements of 10.5% in mIoU, 8.6% in mP, 5.8% in mR and 7.4% in mF1 metrics. The slight decrease in speed, compared to the improvement in segmentation accuracy, is still acceptable.

In summary, through a comprehensive comparison with the other six models in Table 6, CPHNet stands out as the optimal model for pumpkin seedling point cloud stem segmentation, further demonstrating its outstanding performance in this specific task of pumpkin seedling point cloud stem segmentation.

### 3.6. Visualize Results Comparison

For a more intuitive understanding of CPHNet’s superiority in the task of pumpkin seedling point cloud stem segmentation, we selected three representative point cloud data of pumpkin seedlings and visualized the segmentation results of the models listed in Table 6. The visualizations are presented in Table 7. The visualization displays the pumpkin seedlings segmented into two categories: the green region represents the stem, whereas the blue region represents other parts.

In Group A, we selected pumpkin seedling point cloud data with multiple branches to observe the segmentation results, evaluating the network’s performance on various forms of pumpkin seedling point cloud data. It is evident that apart from CPHNet, the results segmented by other models more or less incorrectly labeled the branches as stems. This indicates that CPHNet outperforms the other six models in adapting to the different forms of pumpkin seedling stems.

In Group B, we selected pumpkin seedling point cloud data with multiple potted planters to observe the segmentation results, simulating the segmentation performance of the network model in the presence of interference from other objects. By comparing the visualization of the segmentation results of these six models, we observed that compared to the PointNet series models without attention mechanisms, GACNet, CSANet and CPHNet with attention mechanisms made fewer errors in mislabeling parts as stems. Among them, CPHNet segmented the stems most completely and with smoother segmentation edges.

In Group C, we selected pumpkin seedling point cloud data with various types of noise, including walls and outliers, to observe the segmentation results, simulating the performance of the network model in segmenting pumpkin seedling point cloud data stems in complex scenarios. We observed that CPHNet still performed the best. Although PointNet++ and CSANet segmented the stems quite completely, they were both affected by noise, incorrectly labeling some outliers and planter bodies as stems.

In Group D, we selected point cloud data containing two pumpkin seedlings simultaneously to observe the segmentation results, simulating the network model’s performance on pumpkin seedling point cloud stem segmentation in a scenario involving multiple plants. Due to significant occlusion and overlapping areas among multiple pumpkin seedling point clouds, segmentation presented substantial challenges. In the segmentation results, all models exhibited some instances of minor mislabeling. However, overall, CPHNet outperformed the other six models. Not only did CPHNet successfully segment the stems of both pumpkin seedlings, but it also provided the most complete segmentation of the stems.

Overall, CPHNet performs the best. The reasons behind CPHNet’s superior segmentation performance over other models are as follows: (a) Utilization of CRA-MLP in the local feature-extraction phase enhances the network’s perception and discrimination of local features, effectively filtering noise from feature information. (b) Integration of PESA in the global feature aggregation phase dynamically adjusts the importance of features by learning the weight relationships between each feature and others, thereby accommodating different shapes of pumpkin seedling stems. (c) The use of HCE-Dice Loss in the training process provides comprehensive feedback, enabling the model to focus on both classification and target shape matching, resulting in smoother segmentation edges.

### 3.7. Application and Prospect

To assess the effectiveness of the CPHNet model in actual pumpkin seedling point cloud stem segmentation, initially, we used the RVC-X-mini structured camera to capture real-time point cloud data of pumpkin seedlings at the Institute of Facility Agriculture, South China Agricultural University, in Guangdong Province. Subsequently, after processing the data, we input it into the CPHNet. The input point cloud data (typically N points, each with a feature dimension of D = 3) first undergo feature extraction through multiple layers of perceptrons (*MLP*) to enhance the feature representation capability of each point. The overall structure of the model is similar to U-Net, comprising encoder and decoder parts. The encoder uses multiple Set Abstraction (SA) blocks to hierarchically sample point cloud features. These SA blocks progressively reduce the number of points (N→N/4→N/16→N/64→N/256) while increasing the feature dimension per point (64→128→256→512→1024). In the local feature-extraction stage, CPHNet introduces the CRA-MLP module to enhance the network’s perception of local features. This module replaces standard *MLP*s and focuses more on task-relevant features during feature fusion, thereby improving fusion accuracy. For global feature integration, CPHNet employs the PESA module. This module dynamically adjusts the importance of features by learning weight relationships between them, enhancing the network’s ability to integrate global information and improving generalization and segmentation accuracy. The model also utilizes a specific loss function, HCE-Dice Loss, which combines cross-entropy and Dice loss. This loss function simultaneously focuses on pixel-level classification and target shape matching, ensuring precise segmentation of different parts of the pumpkin seedling stem.

**Figure 6:**
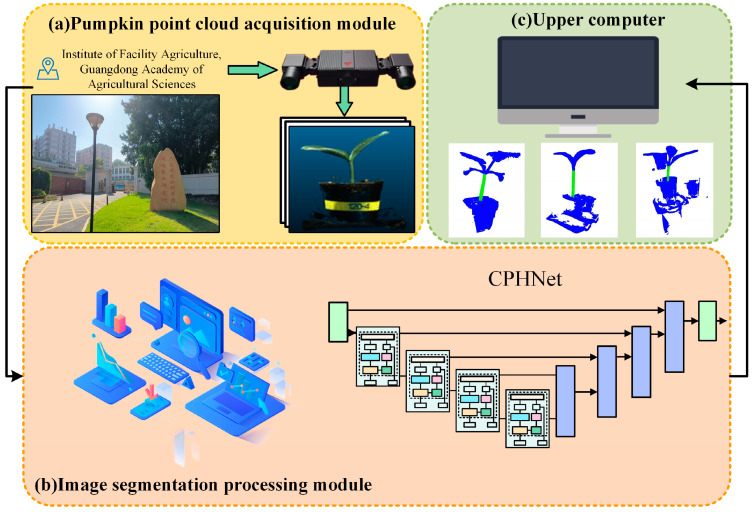
Workflow for a practical application.

The resulting application results were finally displayed on the monitor. Figure 6 illustrates the associated workflow.

**Figure 7:**
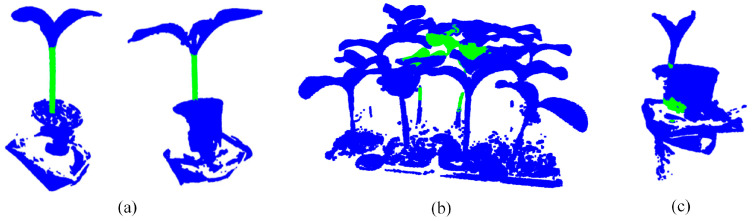
Application results.

From the visualization of the application results in Figure 7a, it is evident that CPHNet demonstrates robust performance in most cases, accurately segmenting the stem portion of the pumpkin seedling point cloud data. This is sufficient evidence to establish the stability of CPHNet in real-world scenarios. However, there are still instances where the results are less than ideal. Analyzing these samples can provide valuable insights for further enhancing the network’s performance. In (b), we input a clustered pumpkin seedling point cloud data, it is evident that only a small portion of the stems has been segmented, and some leaves have been mistakenly classified as part of the stem. This is due to the absence of such samples in the training set and the considerable occlusion between pumpkin seedlings in the clustered pumpkin seedling point cloud data, which further reduces the segmentation effectiveness. In our subsequent research, we aim to collect point cloud data of these clustered pumpkin seedlings to expand the application scope of CPHNet, enabling it to handle clustered pumpkin seedling data and further enhance the efficiency of practical segmentation. In (c), we input point cloud data from the early stage of pumpkin seedling growth. It can be observed that only a small portion of the stem is segmented, with some parts of the pot mistakenly segmented as the stem. At this growth stage, the pumpkin seedling’s leaves have not fully expanded, resulting in a short length of the stem. Consequently, there is a significant imbalance in the label count between the stem class and other classes of points. Moreover, the dataset contains a limited quantity of point cloud data for pumpkin seedlings at this growth stage, contributing to deviations in the segmentation results for different growth stages of pumpkin seedlings. In our future research, we aim to balance the quantity of point cloud data for different growth stages of pumpkin seedlings and further adjust the model’s classification component to balance the model’s sensitivity to each category.

To truly make this network model significantly impactful in real-life scenarios, there are still some issues that need to be addressed. To further enhance the applicability and practicality of CPHNet, the following improvements will be made: (1) The performance of the network model is closely linked to the size and precision of the dataset. However, compared to the collection and annotation of 2D images, acquiring and annotating point clouds in reality is time-consuming and prone to errors. Therefore, in the future, we aim to employ different 3D data collection tools to gather higher-precision 3D point cloud data of pumpkin seedlings. Additionally, to reduce the manual cost of data annotation, we may consider using data synthesis techniques via simulators to augment the pumpkin seedling 3D point cloud data for network training. Lastly, the design of more suitable deep learning network models for handling 3D plant structures will further enhance the segmentation effectiveness and efficiency of pumpkin seedling 3D point clouds, catering to real-time requirements in specific agricultural scenarios. (2) Increase post-segmentation data processing, such as accurately calculating the specific length of segmented stems, and providing more detailed data support for predicting the growth status of pumpkin seedlings. (3) There is a growing demand for real-time applications in actual agricultural production. Hence, there will be a focus on designing and optimizing network models to meet the real-time requirements and specific scenarios in agriculture. By improving the algorithm’s computational efficiency and response speed, it will handle large-scale plant 3D point cloud data more rapidly, meeting real-time demands in various farm-management scenarios. Simultaneously, considering the application in mobile devices and embedded systems, the optimization of models to run efficiently in resource-constrained environments will better serve the practical application scenarios of agricultural production.

## 4. Discussion

We compared CPHNet with six other point cloud segmentation networks: PointNet, PointNet++, PointNet++ (MSG), GACNet, PointNeXt and CSANet. Table 7 presents the segmentation visualization results of these seven networks. As shown in Table 6, our proposed model, CPHNet, outperforms the other six models in different indicators such as mIoU, mP, mR and mF1. PointNet directly processes raw point cloud data without requiring preprocessing. It independently processes each point and uses max pooling to aggregate global features. By combining global features with local features of each point, it generates segmentation results for each point, preserving the geometric information of the point cloud and avoiding complex preprocessing steps. However, its various indicators are only 64.1%, 68.3%, 72.5%, 70.3%. On the other hand, PointNet++ uses grouping and sampling methods to extract local features and captures geometric information at different scales, enhancing adaptability to point cloud regions of varying sizes and densities. The hierarchical structure extracts features layer by layer and aggregates global features, improving computational efficiency and feature representation capabilities. Its metrics are 79.9%, 84.5%, 89.8%, 87.0%. PointNet++ (MSG) employs Multi-Scale Grouping (MSG) to extract features at multiple scales, capturing geometric information more comprehensively. This multi-scale feature extraction enables the model to handle complex shapes with higher accuracy and robustness. However, while multi-scale feature extraction helps in capturing intricate geometric shapes, sometimes local features may not fully describe extremely complex or unique shapes, leading to a decrease in segmentation accuracy. Its metrics are 80.8%, 84.0%, 91.2%, 87.5%. GACNet introduces a graph attention mechanism, allowing it to compute attention weights within local neighborhoods and more accurately capture local and global features of the point cloud data. It calculates edge weights between each central point and its neighboring points, enhancing the model’s ability to segment boundaries and complex structures. Its metrics are 75.7%, 80.2%, 87.2%, 83.5%. Compared with PointNeXt, PointNeXt introduces receptive field extension and model extension strategies. It uses reverse residual *MLP* (InvResMLP) blocks to improve feature extraction and mitigate the gradient disappearance problem. These blocks combine separable *MLPS* and residual joins to enhance the accuracy of model segmentation. However, the model does not introduce attention mechanism, which makes it difficult to dynamically adjust the importance of features when dealing with complex pumpkin seedling stem structure. Compared to the model, our model has improved +2.7%, +1.6%, +1.5% and +1.6%. Finally, compared with CSANet, CSANet uses cross-self-attention mechanism to interact coordinate information and feature information of point cloud and dynamically adjust the importance of features. Through interactive extraction, the model can better capture the spatial relationship and geometric features in the point cloud data, so as to improve the accuracy and robustness of feature extraction. Its various indicators are 88.0%, 91.0%, 93.7% and 92.3%.

In the visualization in Table 7, the pumpkin seedlings are divided into two categories: the green areas represent the divided stem sections, while the blue areas represent the other sections, which allows us to more intuitively understand the segmentation of each network. Comparing the metrics in Table 6 and the segmentation visualization results in Table 7 indicates that, except for CPHNet, more or less branches in the pumpkin seedlings are incorrectly labeled as stems in the visualization results of the other six network segmentation. Compared to the other networks, CPHNet demonstrates the best segmentation capability for the stem parts of pumpkin seedlings, indicating that CPHNet is superior to the other six models in adapting to different forms of pumpkin seedling stems.

## 5. Conclusions

To explore the optimal method for segmenting pumpkin seedling stem parts in point cloud data, we proposed a pumpkin seedling stem segmentation algorithm based on CPHNet. First, in the local feature-extraction phase, we introduced a CRA-MLP module to enhance the network’s perception and discrimination of local features. This aimed to prioritize features that are more representative of the current task, thereby enhancing the network’s representation capability and effectively filtering noise from feature information. Next, in the global feature integration phase, we introduced PESA, which dynamically adjusts the importance of features by learning the weight relationships between each feature and others. This adjustment facilitates better capture of internal information and dependencies to adapt to various shapes in pumpkin seedling point cloud data. Lastly, in the loss function part, we introduced HCE-Dice Loss, which simultaneously focuses on pixel-level classification and target shape matching to better learn accurate segmentation of different components. This provides more comprehensive feedback during the training process. The experimental results demonstrate that this method overcomes the missegmentation issues in pumpkin seedling stem part segmentation in point cloud data, adapting better to various shapes in the pumpkin seedling point cloud data, and enhancing the model’s segmentation accuracy and generalization. CPHNet improves the accuracy of the PointNet++ model in pumpkin seedling stem part segmentation, better matching the data features of pumpkin seedling point clouds during training. This research provides more accurate and reliable means for pumpkin agricultural production management. Precise segmentation of pumpkin seedling stems aids in the timely detection and identification of diseases or abnormal growth, providing farmers with effective decision-making criteria to take measures against disease spread or abnormal growth proliferation, ultimately enhancing pumpkin yield and quality. Additionally, precise segmentation provides a reliable data foundation and technical support for the development of modern agricultural technologies such as intelligent agriculture, automated cultivation and agricultural robotics. In the experimental section, we divided the collected 1192 pumpkin seedling point cloud data into training and testing sets at a ratio of 4:1. Under these conditions, CPHNet achieved a mIoU of 90.4%, mP of 93.1%, mR of 95.6%, mF1 of 94.4% and a speed of 0.03. Compared to the traditional PointNet++ method, CPHNet improved mIoU by 10.5%, mP by 8.6%, mR by 5.8% and mF1 by 7.4%. It significantly increased segmentation accuracy within an acceptable speed range. The results indicate that CPHNet outperforms other test models, validating the proposed methods’ effectiveness in achieving more accurate and stable segmentation of pumpkin seedling stem parts from point cloud data.

## Figures and Tables

**Table 1:** Data preprocessing.

Pre-Statistical Filtering	After Statistical Filtering	Primary Point Cloud	Random Rotation	Random Jitter	Gaussian Noise
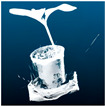 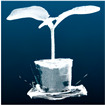	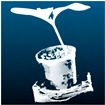 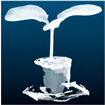	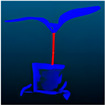 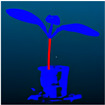	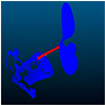 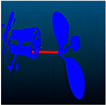	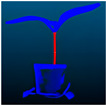 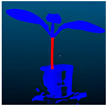	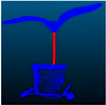 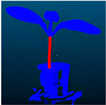

**Table 2:** Software and hardware environment settings.

Hardware environment	CPU	AMD Ryzen 7 5800H with Radeon Graphics
	RAM	16 GB
	GPU	NVIDIA GeForce RTX 3060
	Video memory	6 GB
Software environment	OS	Windows11
	CUDA	11.2
	Python	3.9.13
	Torch	2.0.0

**Table 3:** Module effectiveness experiments.

Module	Method	mIoU	mP	mR	mF1	Params (M)	Speed
Data processing	Raw data	87.7	89.0	95.6	92.2	7.282	0.03
	+Statistically filtered	89.3	92.1	94.7	93.4	7.282	0.03
	+Statistically filtered +Data enhanced	90.4	93.1	95.6	94.4	7.282	0.03
CRA-MLP	PointNet++	79.9	84.5	89.8	87.0	1.403	0.10
	+CRA-MLP	84.4	87.0	97.1	91.8	3.443	0.03
	ReLU	83.8	86.3	94.0	90.0	3.443	0.03
	PReLU	84.4	87.0	97.1	91.8	3.443	0.03
PESA	PointNet++	79.9	84.5	89.8	87.0	1.403	0.10
	+SA	81.7	84.6	92.9	88.5	6.591	0.03
	+PESA	84.3	87.5	93.1	90.2	6.583	0.03
HCE-Dice Loss	Cross Entropy Loss	79.9	84.5	89.8	87.0	1.403	0.10
	HCE-Dice Loss	84.6	86.5	97.6	91.7	1.403	0.10

**Table 4:** Ablation experiment of CPHNet.

Group	Method	mIoU	mP	mR	mF1	Params (M)	Speed
A	PointNet++	79.9	84.5	89.8	87.0	1.403	0.10
B	A+CRA-MLP	84.4	87.0	97.1	91.8	3.443	0.03
C	A+PESA	84.3	87.5	93.1	90.2	6.583	0.03
D	A+HCE-Dice Loss	84.6	86.5	97.6	91.7	1.403	0.10
E	B+PESA	87.4	90.3	93.5	91.9	7.282	0.03
F	B+HCE-Dice Loss	87.0	88.3	95.7	91.8	3.443	0.03
G	C+HCE-Dice Loss	86.6	90.1	92.8	91.5	6.583	0.03
H	E+HCE-Dice Loss	90.4	93.1	95.6	94.4	7.282	0.03

**Table 5:** Comparison of visualization results for different modules.

Method	A	B	C	D
Original Point cloud	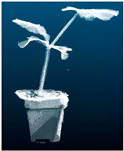	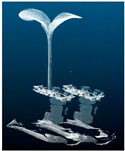	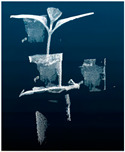	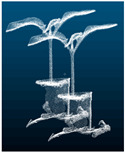
Labeled Point cloud	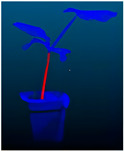	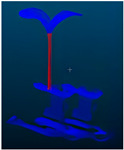	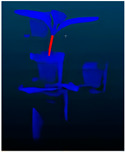	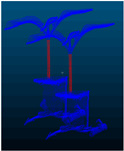
PointNet++	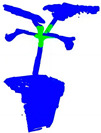	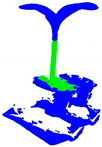	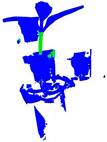	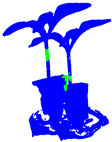
+CRA-MLP	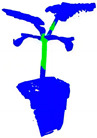	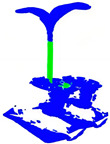	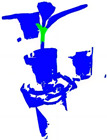	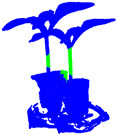
+CRA-MLP+ PESA	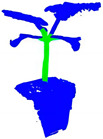	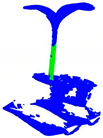	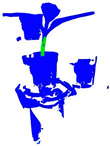	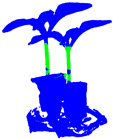
CPHNet	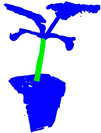	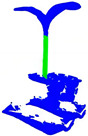	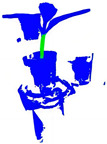	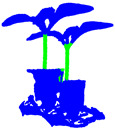

**Table 6:** Compare the performance of CPHNet with SOTA models.

Method	mIoU	mP	mR	mF1	Params(M)	Speed
PointNet	64.1	68.3	72.5	70.3	8.323	0.01
PointNet++	79.9	84.5	89.8	87.0	1.403	0.10
PointNet++(MSG)	80.8	84.0	91.2	87.5	1.734	0.07
GACNet	75.7	80.2	87.2	83.5	1.318	0.04
PointNeXt	87.7	91.5	94.1	92.8	0.982	0.04
CSANet	88.0	91.0	93.7	92.3	15.946	0.05
CPHNet	90.4	93.1	95.6	94.4	7.282	0.03

**Table 7:** Comparison of visualization results for SOTA models.

Method	A	B	C	D
Original Point cloud	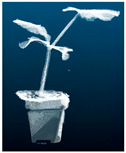	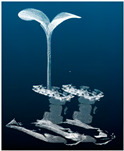	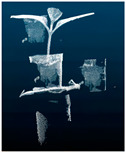	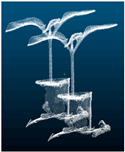
Labeled Point cloud	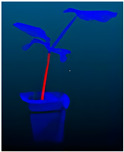	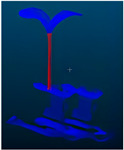	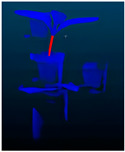	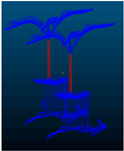
PointNet	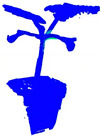	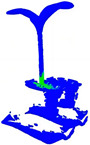	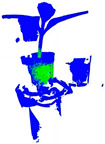	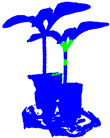
PointNet++	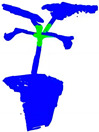	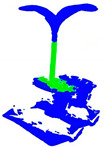	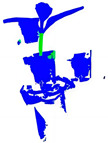	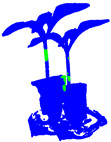
PointNet++(MSG)	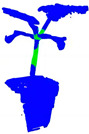	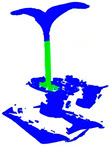	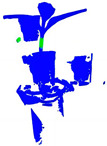	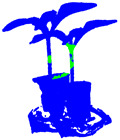
GACNet	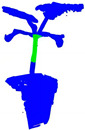	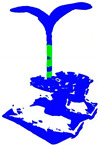	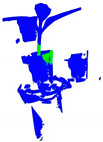	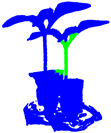
PointNeXt	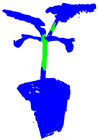	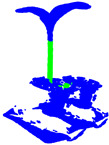	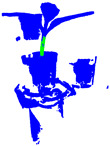	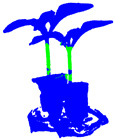
CSANet	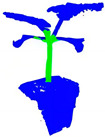	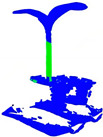	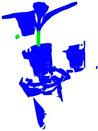	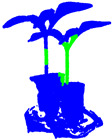
CPHNet	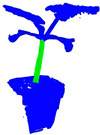	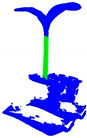	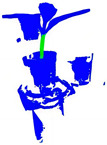	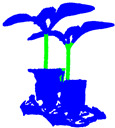

## Data Availability

The partial datasets utilized and examined in this research have been posted on the website https://github.com/ZhouGuoXiong/CPHNet, accessed on 2 December 2023. Furthermore, for access to all bespoke datasets used in this study, please contact the corresponding author.
